# Cereblon-binding proteins expression levels correlate with hyperdiploidy in newly diagnosed multiple myeloma patients

**DOI:** 10.1038/s41408-019-0174-z

**Published:** 2019-01-29

**Authors:** Katharina Kriegsmann, Marc-Andrea Baertsch, Mohamed H. S. Awwad, Maximilian Merz, Dirk Hose, Anja Seckinger, Anna Jauch, Natalia Becker, Axel Benner, Marc S. Raab, Jens Hillengass, Uta Bertsch, Jan Dürig, Hans Jürgen Salwender, Mathias Hänel, Roland Fenk, Markus Munder, Katja Weisel, Carsten Müller-Tidow, Hartmut Goldschmidt, Michael Hundemer

**Affiliations:** 1 Department of Hematology, Oncology and Rheumatology, Heidelberg University, Heidelberg, Germany; 2 National Center for Tumor Diseases, University Hospital, Heidelberg, Germany; 3 Institute of Human Genetics, University Heidelberg, Heidelberg, Germany; 4 Division of Biostatistics, German Cancer Research Center, Heidelberg, Germany; 5 Department of Hematology, University Essen, Essen, Germany; 6 Asklepios Klinik Hamburg Altona, Hamburg, Germany; 7 Klinikum Chemnitz, Chemnitz, Germany; 8 Department of Hematology, Oncology and Clinical Immunology, University Düsseldorf, Düsseldorf, Germany; 9 Department of Hematology, Oncology, and Pneumology, University Medicine Mainz, Mainz, Germany; 10 Department of Hematology, Oncology, Immunology and Rheumatology, University Tübingen, Tübingen, Germany

## Abstract

Immunomodulatory drugs (IMIDs) are very effective in the treatment of multiple myeloma (MM). The description of their cereblon-mediated mechanism of action was a hallmark in MM research. Although the importance of IMID-induced degradation of cereblon-binding proteins is well described in vitro, the prognostic value of their expression levels in MM cells is less clear. Based on recently published data showing somewhat conflicting RNA levels, we analyzed the association between the levels of the Ikaros family zinc finger protein 1 (IKZF1), IKZF3, and karyopherin subunit alpha 2 (KPNA2) proteins measured by flow cytometry and prognostic parameters in 214 newly diagnosed MM patients who were randomized in the GMMG HD6 trial. No statistically significant associations between the expression levels and age, gender, light chain type, International Staging System (ISS) stage or cytogenetic high- and normal risk groups could be identified. Hyperdiploid MM cells expressed significantly higher levels of IKZF1, IKZF3 and KPNA2 than nonhyperdiploid cells. In contrast, translocation t(11;14) was associated with significantly lower expression levels. In conclusion, the observed overexpression of cereblon-binding proteins in MM cells with gain of chromosomes 5, 9, 11, 15, and 19 is consistent with the previously proposed positive regulation of MYC by IKZF1 and IKZF3, as well as MYC activation in hyperdiploid MM cells.

## Introduction

Lenalidomide, an immunomodulatory drug (IMID), is a highly effective treatment option for patients with newly diagnosed and relapsed multiple myeloma (MM) and achieves response rates of up to 70% in combination with dexamethasone^1–3^.

In terms of its mechanism of action, lenalidomide binds to cereblon (CRBN), the substrate adaptor of the CRL4^CRBN^ E3 ubiquitin ligase complex^4^. By modulating the substrate specificity of the CRL4^CRBN^ E3 ubiquitin ligase, lenalidomide induces the selective ubiquitination and subsequent degradation of Ikaros family zinc finger protein (IKZF) 1 and IKZF3 in MM cells^5,6^. IKZF3 induces the expression of interferon regulatory factor 4 (IRF4) and IRF4 is essentially involved in the positive feedback regulation of the MYC oncogene^7^. Consequently, proteasomal degradation of the transcription factors IKZF1 and IKZF3 leads to MM cell death.

The expression levels of several CRBN-binding proteins, including IKZF1, IKZF3, and karyopherin subunit alpha 2 (KPNA2), are associated with clinical variables and cytogenetic aberrations and have predictive and prognostic relevance in IMID-treated patients with relapsed or newly diagnosed MM^8–10^. Notably, in the study by Krönke et al., IKZF1 mRNA was expressed at higher levels in patients with newly diagnosed International Staging System (ISS) stage III MM than in patients diagnosed with ISS stage I and II MM; lower IKZF1 and IKZF3 expression was detected in patients with gains of 1q21. In this study, a high pretreatment IKZF1 RNA level was identified as an adverse prognostic factor for progression-free survival (PFS). IKZF1 expression was not correlated with the response to induction therapy in patients who received lenalidomide and intensive chemotherapy^8^. In contrast, Zhu et al. have shown that low IKZF1 expression levels are an adverse prognostic factor for the response and survival of patients with relapsed/refractory MM treated with an IMID-based therapy using gene expression profiling^10^. Similarly, in the study by Pourabdollah et al., low IKZF1 and IKZF3 levels correlated with shorter PFS and overall survival (OS), as evidenced by immunohistochemistry of bone marrow from patients with relapsed/refractory MM who were treated with lenalidomide^9^. Sehgal et al. did not identify correlations between baseline immunohistochemical staining for the IKZF1 and IKZF3 proteins in tumor cells and the response to IMIDS or survival^11^. Moreover, Zhu et al. noted the prognostic relevance of mRNA levels of KPNA2, a nuclear transport protein that has been associated with B-cell development, in plasma cells (PCs)^12^. In patients with MM treated with IMIDs, a high KPNA2 expression level was associated with shorter OS^10^.

These current studies have therefore yielded contradictory results regarding the prognostic value of IKZF1 and IKZF3 expression in MM cells, possibly because they were performed in relatively small patient cohorts. The aim of our current study was therefore to determine the expression levels of the CRBN-binding proteins IKZF1, IKZF3, and KPNA2 in MM cells by flow cytometry and to assess correlations of their expression levels with clinical and prognostic factors measured at the time of diagnosis in a large patient cohort.

## Material and methods

### Patient selection and data matching

An analysis of 214 patients who were newly diagnosed with MM and randomized to participate in the multicenter prospective phase III trial GMMG-HD6 on the “Effect of Elotuzumab in VRD (Velcade, Revlimid, and Dexamethasone) Induction/Consolidation and Lenalidomide Maintenance” conducted by the German-Speaking Myeloma Multicenter Group (GMMG) was performed. Clinical parameters, including age, gender, and serum heavy and light chain type by immunofixation, and ISS were assessed as a part of the routine clinical examination. The cytogenetic examinations were performed in a central lab. All patients provided written informed consent before participating in the study. Approval was obtained by the ethics committee of the University of Heidelberg in collaboration with the participating ethics committees.

### Molecular cytogenetic testing

Molecular cytogenetic testing was performed using a previously described method^13^. Briefly, CD138^+^ bone marrow PCs were purified using auto-magnetic-activated cell sorting with anti-CD138 immunobeads as published^14^. For interphase fluorescence in situ hybridization analyses, a panel of two-color probe sets was used to detect numerical changes at the chromosomal loci 1q21/13q14, 5p15/5q35, 8p21/19q13, 9q34/15q22, and 11q22.3/17p13 as well as the IgH-translocations t(11;14)(q13;q32), t(4;14)(p16;q32), t(14;16)(q32;q23), or any other IgH-rearrangement. Hybridization was performed according to the manufacturer’s instructions (MetaSystems, Altlussheim, Germany and Cytocell, Cambridge, UK) and a minimum of 100 interphase nuclei per probe were evaluated using an automated spot counting system (Applied Spectral Imaging, Edingen-Neckarhausen, Germany). Hybridization efficiency was validated using interphase nuclei obtained from the bone marrow of a healthy donor, and the thresholds for gains, deletions, and translocations were set at 10%. Hyperdiploidy was defined according to the criteria described by Wuilleme et al., which require trisomy of at least two of three chromosomes: 5, 9, and 15^15^. Two high-risk cytogenetic definitions were applied to facilitate comparisons with the results from other research groups: Neben et al.—the presence of deletion 17p13 and/or translocation t(4;14), gain of 1q21 (>3 copies)^16^, and Krönke et al.—the presence of deletion 17p and/or t(4;14) or t(14;16)^8^.

### Plasma cell immunophenotyping

Plasma cell immunophenotyping was performed using cytoplasmic anti-κ-allophycocyanin (APC, TB28-2), cytoplasmic anti-λ-APC-H7 (1-155-2), CD19-phycoerythrin (PE)-Cy7 (HIB19), CD27-PerCP-Cy5.5 (L128), CD38-PE (HB7), CD45-V450 (2D1), CD56-fluorescein (FITC, NCAM16.2), CD81-APC-H7 (JS-81), and CD138-V500-C (MI15) antibodies (all obtained from BD Biosciences, Heidelberg, Germany). Fixation and permeabilization (FIX&PERM) solutions A and B (Biozol, Eching, Germany) were used for cytoplasmic anti-κ and anti-λ staining, and BD FACS^TM^ Lysing Solution was used to lyse red blood cells (BD, Heidelberg, Germany). Before measurements were obtained, the cells were washed twice and resuspended in phosphate-buffered saline (Life Technologies, Carlsbad, USA). Measurements were performed on a FACSCanto™ II cell analyzer (BD, Heidelberg, Germany). Data were analyzed using the BD FACSDiva™ (BD, Heidelberg, Germany) and Infinicyt™ software packages (Cytognos, Salamanca, Spain). PCs were identified based on the coexpression of CD38 and CD138 antigens. An aberrant PC expression profile was defined as CD45-low/negative, CD56-positive, CD19-negative, and light chain-restricted^17,18^. CD27 and CD81 were evaluated separately as prognostic markers. The cutoff for the level of positive CD27 and CD81 antigen expression was defined as 20% compared to negative controls.

**Fig. 1:**
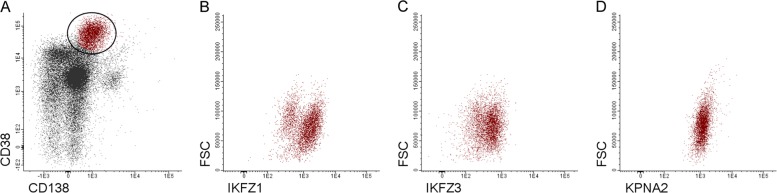
Gating strategy used to identify CRBN-binding proteins in MM cells. Plasma cells (PCs) were identified as CD38/CD138-positve cells among leukocytes (**a**). The expression levels of IKZF1, IKZF3 and KPNA2 were determined by measuring the absolute median fluorescence intensity of the whole PC population (**b**, **c**, **d**; FSC, forward scatter)

Similarly, the expression of CRBN-binding proteins was detected by flow cytometry according to the antibody combinations and the protocol for cytoplasmic staining listed in Table 1. IKZF1, IKZF3, and KPNA2 expression in PCs (CD138^+^, CD38^+^) was determined by measuring the fluorescence intensity (FI, Fig. 1).

**Table 1:** Antibody panel used in flow cytometry assays to identify cereblon-binding proteins in myeloma cells

Fluorochrome	V450	V500-C	Alexa Fluor488/FITC	PE	PerCP	APC
Control tube (clone, company)	/	CD138 (MI15, BD)	/	/	/	CD38 (MHCD3805, Life Technologies)
Tube 1 (clone, company)	CD45 (2D1, BD)	CD138 (MI15, BD)	IKZF1 (R32–1149, BD Pharmingen)	IKZF3 (S50–895, BD Pharmingen)	CD56 (5.1H11, BioLegend)	CD38 (MHCD3805, Life Technologies)
Tube 2 (clone, company)	CD45 (2D1, BD)	CD138 (MI15, BD)	KPNA2 (orb188808, Biorbyt)	/	CD56 (5.1H11, BioLegend)	CD38 (MHCD3805, Life Technologies)

### Statistical analysis

For descriptive statistics, categorical data are presented as absolute numbers and percentages, and continuous data are presented as medians and ranges. The nonparametric Kruskal−Wallis rank sum test was used to test for differences in location of the distribution of continuous variables (IKZF1, IKZF3, and KPNA2 FI in PCs) with respect to categorical variables with two or more categories (gender, light chain type, ISS stage, cytogenetic risk category, CD27 and CD81 expression, and single cytogenetic abnormalities). Age was considered as a continuous variable and a univariate linear regression model was used to investigate the impact of age on log-transformed median FI values variables (IKFZ1, IKZF3, and KPNA2). All investigations were provided in an exploratory manner. Therefore, no correction for multiple testing was done. A two-sided significance level of 5% was used for all statistical analyses. All statistical analyses were performed with the statistical software environment R, version 3.3.2 (R Development Core Team).

## Results

### Patient characteristics

Bone marrow samples from 214 patients (129 males and 85 females) with newly diagnosed MM patients, randomized in the GMMG HD6 trial, were analyzed. The median age was 60 (range 37–70) years. ISS stage I, II, and III were observed in 80 (37.4%), 81 (37.9%), and 53 (24.8%) patients, respectively. Depending on the definition (Neben et al. or Krönke et al.), high-risk cytogenetic abnormalities were detected in 40 (20.5%) and 46 (23.6%) patients, respectively. MM cells from 7 patients (3.4%) were negative for CD27, whereas CD81-positive MM cells were detected in 140 patients (69.3%). The demographic and clinical parameters are summarized in Table 2.

**Table 2:** Clinical characteristics and prognostic factors

Patient number, *n*	214
Age, years	60 (37–70)
Gender, *n* (%)	
Male	129 (60.3)
Female	85 (39.7)
Heavy chain type, *n* (%)	
IgG	136 (63.6)
IgA	30 (14.0)
Other (IgM, IgD, and IgE)	4 (1.9)
None	44 (20.6)
Light chain type, *n* (%)	
κ	146 (68.2)
λ	68 (31.8)
β2-microglobulin (mg/l)	3.4 (1.0–17.4)
Albumin (g/l)	37 (20.0–56.0)
ISS, *n* (%)	
I	80 (37.4)
II	81 (37.9)
III	53 (24.8)
Cytogenetic risk group^a^, *n* (%)	
Standard risk	155 (79.5)
High risk	40 (20.5)
Missing	19
Cytogenetic risk group^b^, *n* (%)	
Standard risk	149 (76.4)
High risk	46 (23.6)
Missing	19
Immonophenotypic prognostic markers, *n* (%)	
CD27-positive	196 (96.5)
CD27-negative	7 (3.4)
Missing	11
CD81-positive	140 (69.3)
CD81-negative	62 (30.7)
Missing	12

Unless indicated otherwise, data are presented as medians (ranges)

*ISS* International Staging System

^a^Cytogenetic high-risk group: presence of del17p and/or t(4;14) or a gain at 1q of >3 copies

^b^Cytogenetic high-risk group: presence of del17p and/or t(4;14) or t(14;16)

### IKZF1, IKZF3, and KPNA2 expression in MM cells

**Fig. 2:**
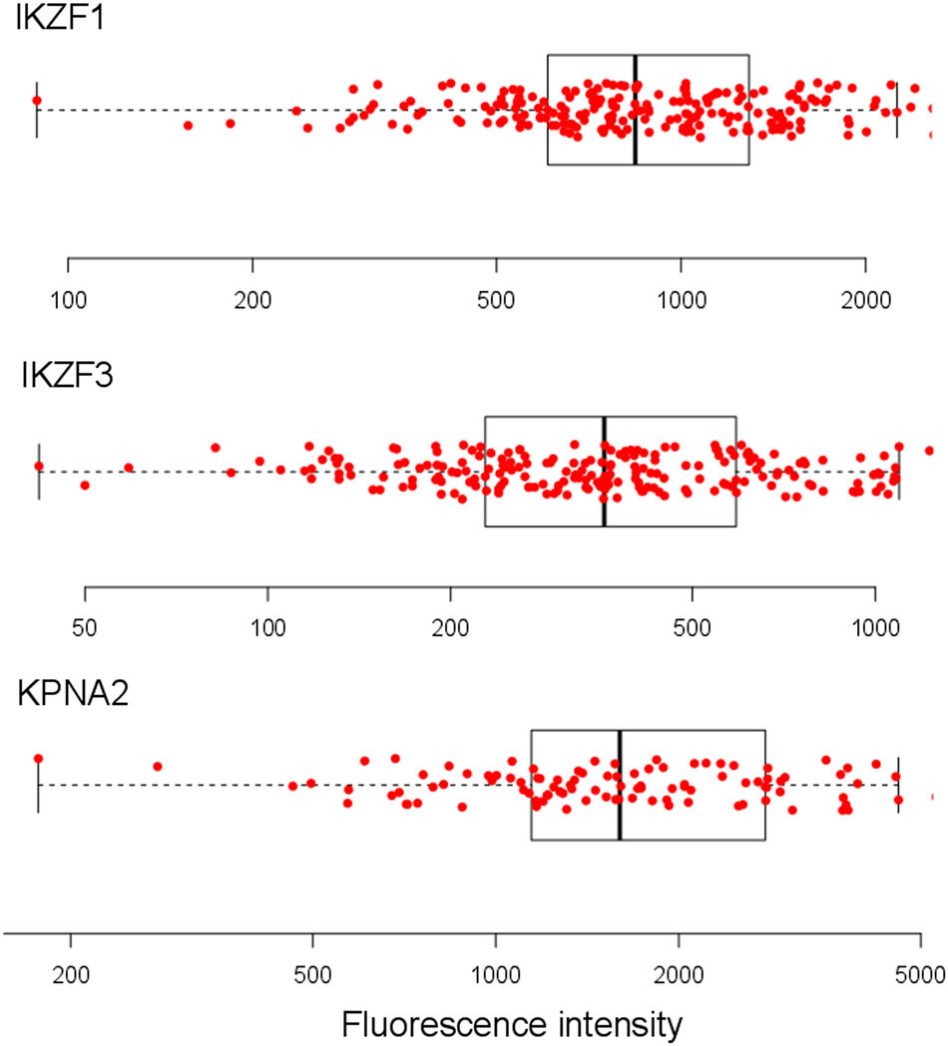
Distribution of IKZF1, IKZF3, and KPNA2 expression in MM cells. IKZF1 (*n* = 214), IKZF3 (*n* = 214), and KPNA2 (*n* = 108) fluorescence intensity was determined on the whole plasma cell population in all patients. Each dot represents a single patient. The boxplots in the background show the median, the first and the third quartiles as well as the lower and upper whiskers

The levels of IKZF1, IKZF3, and KPNA2 expression were determined by flow cytometry. The distribution of the expression levels in myeloma cells for the whole cohort is shown in Fig. 2.

The main focus of the study was to assess whether the expression levels of the individual CRBN-binding proteins differ between clinical and prognostic subgroups (Table 3). No statistically significant differences in IKZF1, IKZF3, and KPNA2 expression levels were identified with regard to age, gender, light chain type, ISS stage, and cytogenetic risk groups.

**Table 3:** IKZF1, IKZF3, and KPNA2 expression levels in MM cells according to clinical and prognostic subgroups

Variable			IKZF1 expression level		IKZF3 expression level		KPNA2 expression level	
		*n*	Median	*P* value	Median	*P* value	Median	*P* value
Age, 10-year increase		214	60	0.06	60	0.32	60	0.98
Gender								
Male		129	844	0.94	341	**0.03**	1582	0.8
Female		85	841		380		1678	
Light chain type								
κ		146	854	0.26	367	0.097	1647	0.33
λ		68	799		337		1600	
ISS								
I		80	797		341		1368	
II		81	832	0.69	355	0.11	1594	0.5
III		53	886		406		1809	
Cytogenetic risk group^a^								
Standard risk		155	850	0.53	357	0.8	1588	0.99
High risk		40	872		371		1695	
Cytogenetic risk group^b^								
Standard risk		149	844	0.66	355	0.5	1577	0.79
High risk		46	914		379		1702	
Cytogenetic aberrations								
gain 1q21	Yes	66	866	0.54	382	0.33	1702	0.87
	No	132	842		358		1579	
gain 1q21 > 3	Yes	16	929	0.65	505	0.07	1691	0.77
	No	182	847		356		1582	
gain 5p15	Yes	87	1057	**<0.001**	403	**0.0013**	1881	**0.0026**
	No	98	745		321		1371	
gain 5q35	Yes	85	1075	**<0.001**	403	**0.0013**	1798	**0.026**
	No	100	745		327		1458	
gain 5	Yes	82	1096	**<0.001**	404	**<0.001**	1810	**0.01**
	No	103	748		321		1456	
del 8p21	Yes	48	1052	0.14	417	0.074	1939	0.085
	No	138	802		336		1520	
gain 9q34	Yes	109	1018	**<0.001**	366	0.057	1735	**0.035**
	No	78	745		337		1308	
gain 11q23	Yes	111	998	**0.015**	366	0.34	1551	0.7
	No	87	798		359		1702	
del 13q14	Yes	100	811	0.14	358	0.83	1577	0.18
	No	98	976		378		1798	
gain 15q22	Yes	100	1015	**<0.001**	372	**0.041**	1761	**0.044**
	No	87	762		334		1492	
gain 19q13	Yes	100	1037	**<0.001**	402	**0.0023**	1881	**0.01**
	No	86	725		304		1408	
del 17p13	Yes	24	823	0.38	362	0.77	2044	0.37
	No	174	854		359		1582	
t(4;14)	Yes	19	858	0.48	377	0.8	1459	0.21
	No	177	850		359		1606	
t(6:14)	Yes	3	646	0.26	325	0.85	2345	0.49
	No	184	847		357		1575	
t(11;14)	Yes	46	646	**<0.001**	225	**<0.001**	1064	**0.0062**
	No	144	1019		388		1712	
t(14;16)	Yes	7	1075	0.51	568	0.21	1789	0.58
	No	190	850		359		1588	
Hyperdiploidy	Yes	94	1033	**<0.001**	384	**0.0049**	1951	**0.0023**
	No	93	762		321		1334	
Immunophenotypic prognostic markers								
CD27	Positive	196	807	0.5	357	0.39	1579	0.42
	Negative	7	959		307		1215	
CD81	Positive	140	801	0.59	357	0.97	1432	**0.046**
	Negative	62	997		364		1919	

Median expression levels and *P* values are presented for clinical and prognostic subgroups. The *P* values for categorical prognostic factors are calculated by the Kruskal−Wallis test. For the continuous values of age the linear regression was used. Bold P values indicate statistical significance. KPNA2 expression levels were measured in 108 patients

A gain at chromosome 5 was considered positive when a gain at both 5p15 and 5q35 was present

Hyperdiploidy was defined as trisomy of at least two of three chromosomes: 5, 9, and 15

*del* deletion, *ISS* International Staging System

^a^Cytogenetic high-risk group: presence of del17p and/or t(4;14) and/or a gain at 1q of >3 copies

^b^ Cytogenetic high-risk group: presence of del17p and/or t(4;14) and/or t(14;16)

**Fig. 3:**
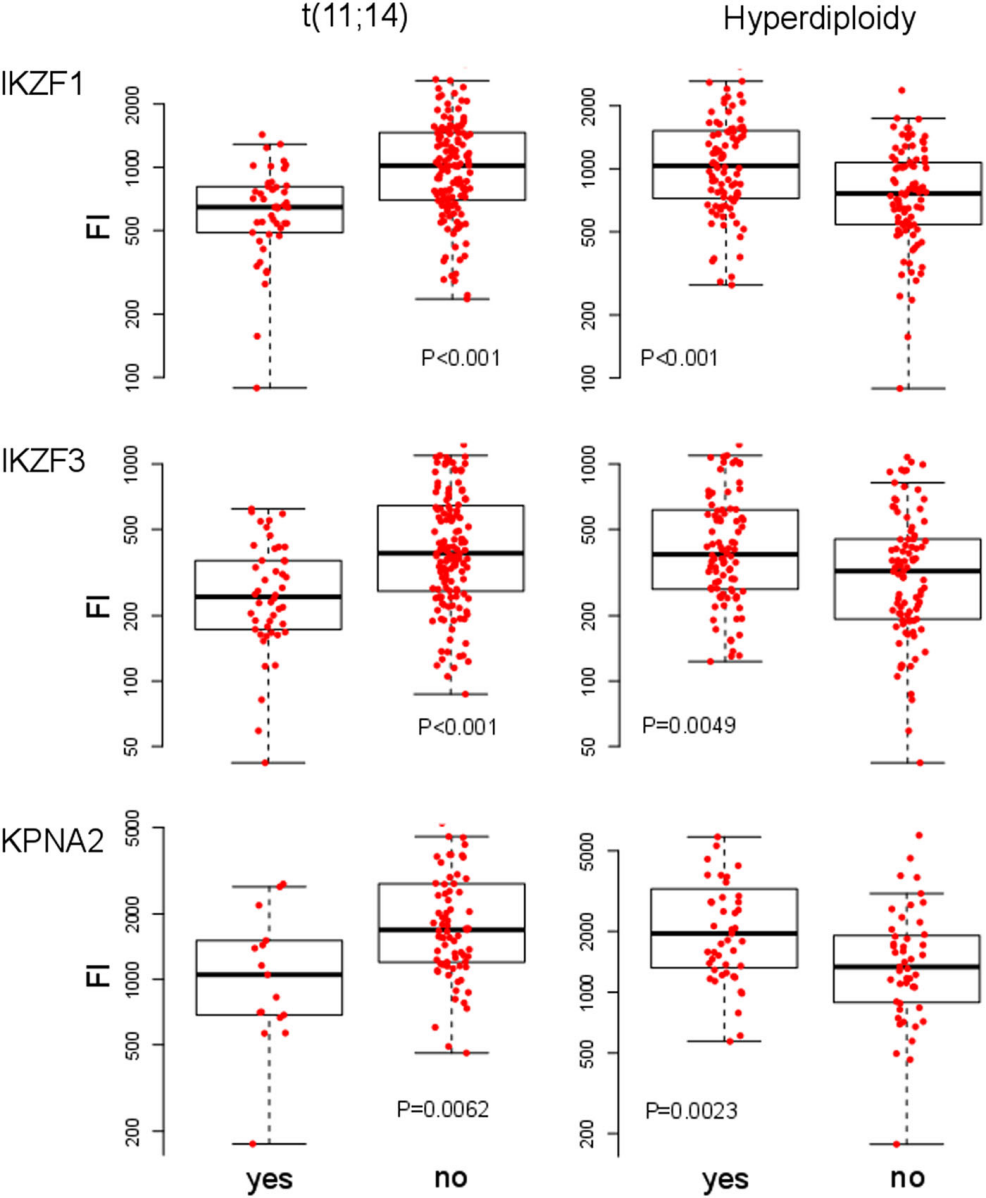
Distribution of IKZF1, IKZF3, and KPNA2 expression in MM cells among cytogenetic aberration subgroups. The figure shows the fluorescence intensity of IKZF1, IKZF3, and KPNA2 on the whole plasma cell population in MM patients with (*n* = 46) and without (*n* = 144) translocation t(11;14) and with (*n* = 94) and without (*n* = 93) hyperdiploidy. Each dot represents a single patient. The boxplots in the background show the median, the first and the third quartiles as well as the lower and upper whiskers. MM multiple myeloma

However, we observed a significant increase in IKZF1, IKZF3, and KPNA2 expression in patients with gains at chromosomes 5, 9 and 15, all of which exhibited gain (a borderline significant difference in IKZF3 expression was observed in MM patients with gain of chromosome 9, *P* = 0.057). Accordingly, patients with hyperdiploidy expressed significantly higher levels of IKZF1, IKZF3, and KPNA2 in MM cells compared to patients without hyperdiploidy (Fig. 3). Additionally, patients with gain 19 also expressed significantly higher levels of all three CRBN-binding proteins in MM cells.

Interestingly, in contrast to hyperdiploidy, t(11;14) was associated with significantly lower expression of IKZF1, IKZF3, and KPNA2 in MM cells (Fig. 3).

Regarding the prognostic factors CD27 and CD81, significant differences in KPNA2, but not IKZF1 or IKZF3, expression levels were detected between CD81-positive and -negative MM cells. Compared to CD81-positive MM cells, CD81-negative MM cells showed a higher KPNA2 FI (1919 vs. 1432, *P* = 0.046). No statistically significant differences in the levels of CRBN-binding proteins were observed between patients with CD27-positive and -negative MM cells.

## Discussion

The expression levels of IKZF1, IKZF3, and KPNA2 have predictive and prognostic value in determining the response of patients with MM to IMID treatment. Moreover, an association with clinical variables and cytogenetic aberrations has been proposed^8–10^. In the current study, we determined the expression levels of the CRBN-binding proteins IKZF1, IKZF3, and KPNA2 in MM cells in a cohort of 214 patients with newly diagnosed MM before treatment with an induction therapy containing lenalidomide. A comparison of the protein expression levels in different clinical and prognostic subgroups was performed.

We did not identify any significant differences in IKZF1 and IKZF3 expression with regard to age, gender, ISS stage, and light chain type. These results are consistent with the results reported by Krönke et al., who assessed IKZF1 and IKZF3 mRNA expression levels in PCs from 60 patients with newly diagnosed MM^8^. A high-risk cytogenetic status was defined using the criteria reported by Krönke et al. (presence of del17p and/or t(4;14) or t(14;16)^8^) and criteria applied at our institution (presence of del17p and/or t(4;14) or a gain at 1q21 of >3 copies^16^) to ensure comparability. However, no differences in IKZF1 and IKZF3 expression were identified between cytogenetic standard and high-risk groups using either definition, which also confirms the data reported by Krönke et al. Moreover, we found that del 17p13, del 13q14 and t(4;14) were not associated with higher or lower IKZF1 and IKZF3 expression at the protein level. In contrast, lower IKZF1 expression in patients with a gain at 1q21 was not confirmed in the current study, as similar levels of the IKZF1 protein were detected in MM cells from patients with and without a gain at 1q21.

Analogous results were obtained for the levels of the KPNA2 protein. Although Zhu et al. reported an association between a high KPNA2 RNA expression level and shorter OS in patients with relapsed/refractory MM who were treated with IMIDs^10^, we did not identify an association between the expression of the KPNA2 protein and clinical/prognostic variables.

Genetically, two major groups of MM are distinguished according to ploidy and chromosome 14q32 translocations, hyperdiploid and nonhyperdiploid groups, both of which are assumed to be early mutagenic events^19^. Hyperdiploidy in MM is defined as gain of certain odd-numbered chromosomes and is observed in approximately one-half of patients and, with some exceptions, was described to have a positive prognostic value^20^. A major new finding of the current study is the significant increase in IKZF1, IKZF3, and KPNA2 expression observed in patients with hyperdiploidy defined as trisomy of chromosomes 5, 9, and 15. Similarly, patients with gains at 19q13 expressed significantly higher levels of these proteins, and patients with gain of 11q22.3 expressed higher levels of IKZF1, but not IKZF3 or KPNA2. As described in the study by Wuilleme et al. reviewing large published studies constituting 173 hyperdiploid karyotypes, the most frequent gained chromosomes are the odd-numbered chromosomes: 3, 5, 7, 9, 11, 15, 19, and 21. The use of the combination of chromosomes 5, 9, and 15 is generally the best compromise between specificity and sensitivity for the detection of hyperdiploidy^15^. Therefore, a common underlying mechanism of hyperdiploidy was postulated and was reflected by uniformly increased IKZF1, IKZF3, and KPNA2 expression levels in MM cells with gains at all chromosomes analyzed in the current study (5p15/5q35, 9q34, 11q22.3, 15q22, and 19q13).

MYC has recently been shown to be activated in 67% of myeloma samples and is associated with hyperdiploidy^21,22^. Moreover, kinetic analyses have revealed that IKZF1 and IKZF3 may act as positive regulators of IRF4 and MYC in MM cells, thus providing new understanding of the mechanism of action of IMIDs. As shown in the study by Bjorklund et al., downregulation of MYC led to the sequential downregulation or proteasomal degradation of IKZF1, IKZF3 and IRF4, followed by growth inhibition and apoptosis of MM cells^23^. Our results complement these studies, as we observe increased levels of the IKZF1 and IKZF3 proteins in hyperdiploid MM cells, consistent with the positive regulation and activation of MYC by IKZF1 and IKZF3.

Nonhyperdiploid MM involves translocations of the immunoglobulin heavy chain alleles at chromosome 14q32 with different chromosomes, namely chromosomes 4, 6, 11, 16, and 20^15,24^. In the current study, we observed a significant decrease in the IKZF1, IKZF3, and KPNA2 levels in MM cells with translocation t(11;14), but no differences were observed in cells with other chromosome 14 translocations. Therefore, our current findings differ from the results published by Krönke et al., who did not observe differences in IKZF1 or IKZF3 RNA expression levels in the presence or absence of t(11;14). A prognostic value for t(11;14), which is detected in approximately 15–20% of patients with MM, has not yet been determined^19^, and the obtained results are difficult to interpret in terms of the prognosis or underlying biological mechanism. However, IKZF1, IKZF3, and KPNA2 expression differed in hyperdiploid MM (high protein levels) and t(11;14) MM (low protein levels), highlighting the two major genetically distinguished groups.

Several studies have reported the predictive value of the levels of CRBN-binding proteins regarding the response to IMID therapy and survival outcomes in patients with MM, indicating their considerable potential for use as biomarkers. For example, Zhu et al. reported a lack of clinical response in pomalidomide-treated patients with relapsed/refractory MM who exhibited the lowest quartile range of IKZF1 RNA expression in their MM cells. In this study, cohorts with low IKZF1 and high KPNA2 RNA expression levels showed shorter OS. Moreover, in terms of potential clinical use, the assessment of IKZF1 expression using flow cytometry achieved results consistent with western blot analyses^10^. Bolomsky et al. analyzed IKZF1 expression in MM cells from a cohort of patients with MM who were treated with lenalidomide using flow cytometry and determined correlations with outcomes. High levels of IKZF1 expression on MM cells in patients before treatment were associated with prolonged PFS^25^. Contrary, Dimopoulos et al. could not identify any predictive value of CRBN, IKZF1, and IKZF3 for IMID response in 23 MM patients treated with a lenalidomide-containing regimen and argue against the use of these proteins as predictive biomarkers. In this study, the expression levels of CRBN, IKZF1, and IKZF3 were determined by standard immunohistochemistry in formalin-fixed paraffin embedded bone marrow samples^26^. A correlation between the levels of CRBN-binding proteins in MM cells assessed using flow cytometry and the response of patients with MM to lenalidomide-based induction therapy is the subject of current investigations by our group.

Low CD27 expression was associated with high-risk disease and CD81 positivity was described as an adverse prognostic marker in patients with MM^27–29^. No differences in IKZF1, IKZF3 and KPNA2 expression were observed between CD27-positive and -negative MM cells. On the contrary, KPNA2 expression was increased in CD81-negative MM cells. However, the functional and prognostic importance of this finding remains unclear.

The current study provides the largest scale effort to date, in terms of patient samples analyzed, to characterize the association between CRBN-binding proteins in MM cells and clinical characteristics at first diagnosis. Nonetheless, the present analysis shows several limitations. Compared to other techniques, flow cytometry is a relatively easy-to-establish and fast method to assess protein expression levels and is feasible in large numbers of patients in clinical routine diagnostics. Combined with further antigens it also allows to determine the expression levels of proteins in selected subpopulations of cells (e.g. monoclonal MM cells characterized by light chain restriction). However, no uniform IKZF1, IKZF3 and KPNA2 flow cytometric protocols are available and standardization is warranted. Also, further studies on CRBN-binding proteins discussed in this manuscript relay on other techniques than flow cytometry for expression level measurement. Exemplarily, Dimopoulos et al. use standard immunohistochemistry in formalin-fixed paraffin-embedded bone marrow samples^26^. Krönke et al. and Zhu et al. assess IKZF1, IKZF3 and KPNA2 expression on RNA but not protein level^8,10^. Therefore, comparability of results might be hampered and further studies are required to prove the correlation between different methods.

The current study gives detailed information on the CRBN-binding proteins expression levels in different clinical and prognostic subgroups providing a functional link between previously reported positive regulation of MYC by IKZF1 and IKZF3 and MYC activation in hyperdiploid MM. One could hypothesize that high CRBN-binding proteins expression levels—and therefore hyperdiploidy—might indicate higher dependency on this pathway and IMID treatment might be associated with better response. However, the correlation results and subgroup analyses on CRBN-binding protein expression levels and response to lenalidomide-based induction therapy are still pending and will provide more insights into the predictive value of CRBN-binding proteins levels for IMID treatment.

Moreover, the assessment of cereblon-binding proteins expression in MM cells post lenalidomide treatment would provide additional information on IMID mechanism of action. However, after induction treatment the majority of patients reaches excellent response rates and only few or no MM cells can be detected in the bone marrow of MM patients corresponding to minimal residual disease negativity (MRD). Also, bone marrow assessment post induction (i.e. post lenalidomide) treatment is usually performed in case of serological CR in order to prove MRD status. Therefore, no accessible bone marrow sample with a sufficient number of MM cell to analyze the level of cereblon-binding proteins expression is available post lenalidomide treatment. Hence, cereblon-binding proteins expression levels in MM cells can only be measured at first diagnosis. Future analyses of our group will focus on cereblon-binding proteins expression levels at relapse, therefore providing more insights into resistance mechanism after IMID treatment.

In summary, we demonstrate the feasibility of large-scale IKZF1, IKZF3 and KPNA2 expression level assessment in MM cells by flow cytometry. Regarding cytogenetic abnormalities, high IKZF1, IKZF3, and KPNA2 expression were observed in hyperdiploid MM cells with gain of chromosomes 5, 9, 15, and 19. These results are consistent with the previously proposed positive regulation of MYC by IKZF1 and IKZF3 and MYC activation in hyperdiploid MM. Future correlation analysis of IKZF1, IKZF3, and KPNA2 expression levels and response to lenalidomide-based induction therapy are warranted and will establish the predictive value of CRBN-binding proteins levels.
